# Ruthenium-106 plaque radiotherapy for uveal melanoma: analysis of tumor dimension and location on anatomical and functional results

**DOI:** 10.1186/s12886-022-02521-9

**Published:** 2022-07-16

**Authors:** Reza Mirshahi, Ahad Sedaghat, Ramin Jaberi, Zohreh Azma, Mehdi Mazloumi, Masood Naseripour

**Affiliations:** 1 Eye Research Center, The Five Senses Health Institute, School of Medicine, Iran University of Medical Sciences, Tehran, Iran; 2 Cancer Institute, Imam Khomeini Hospital, Tehran University of Medical Sciences, Tehran, Iran; 3 Radiation Medicine Department, Shahid Beheshti University, Tehran, Iran; 4 Stem Cell and Regenerative Medicine Research Center, Iran University of Medical Sciences, Tehran, Iran

**Keywords:** Uveal melanoma, Brachytherapy, Globe survival, Patient survival, Ru-106 plaque therapy

## Abstract

**Background:**

To report the long-term outcomes of Ru-106 plaque radiotherapy in eyes with uveal melanoma (UM) and to assess the effect of tumor thickness and location on final outcomes.

**Methods:**

Medical records of 234 patients undergoing Ru-106 plaque radiotherapy for UM were reviewed, and the visual outcome, globe preservation, and patient survival were evaluated. The results of 2 groups were compared: 1. between thin (small and medium-sized, thickness < 7 mm, 148 eyes [63.2%]) and thick (thickness ≥ 7 mm, 86 eyes [36.8%]) tumors, and 2. between large (largest basal diameter [LBD] > 12 mm, 109 eyes [46.6%]) and medium/small (LBD ≤ 12 mm, 125 eyes [53.4%]). In addition, a comparison of the juxtapapillary location in 46 eyes (19.7%) versus tumors arising elsewhere and between tumors with and without ciliary involvement in 48 eyes (21.5%) were done.

**Results:**

The patients were followed for a median of 54.2 months (range: 6–194.5 months). After adjusting for baseline visual acuity (VA), there was no significant association between final VA and different dimension and tumor location groups. Final globe preservation was 91.9%, and there was no significant difference between different dimension- and ciliary body involvement groups regarding anatomical success rate. The juxtapapillary tumors had lower globe preservation (80.4% vs .94.7%, *p* = 0.002). The hazard ratio (HR) for enucleation in juxtapapillary tumors was HR = 6.58 (95-CI: 3.84 to 11.21). The overall metastasis rate was 6.8%, with no significant difference in juxtapapillary tumors (4.3% vs.7.4%, *p* = 0.455).

**Conclusions:**

Ru-106 plaque radiotherapy is an effective treatment for thick and large UM. With this type of treatment, the globe preservation rate is lower in juxtapapillary tumors, but there is no significant difference in the metastasis rate.

## Background

There is a long history of uveal melanoma (UM) management, from enucleation to globe-salvaging and sight-preserving methods [1, 2]. A variety of conservative methods have been employed during the last decade, including different types of radiotherapy modalities (plaque radiotherapy, proton beam radiation, and gamma knife radiosurgery), laser therapy (photodynamic therapy, transpupillary thermotherapy), and surgical management (partial lamellar sclerouvectomy, internal resection) [3, 4]. Among these, plaque radiotherapy using different isotopes has become the treatment of choice in most ocular oncology departments. Pioneered in the 1960s by Lommatzsch, the use of Ru-106 applicators as a beta emitter in a variety of sizes and shapes is one of the most commonly used radioactive plaques popularized in the treatment of UM [5]. Although brachytherapy using Ru-106 plaques has been recommended for small to medium-sized tumors up to 5 mm in thickness [6], multiple clinical studies have reported anatomical success with large tumors up to 11 mm in thickness [7, 8]. It is believed that a high basal dose of radiation can be associated with tumoricidal effects through obliterating the tumor blood supply and subsequent necrosis of large UM [9]. This mechanism may be the cause of the successful anatomical results in cases that received total apex radiation less than the recommended dose of 85Gy [8].

Several histopathological and genetic prognostic factors, including cell type, different immunohistochemical markers, and genetic alterations (such as monosomy 3, 8q gain, and *BAP1*mutation), have been reported to be influential in globe and patient survival [10]. However, these factors require tumor biopsy prior to brachytherapy and precise cytogenetic evaluation. Therefore, attempts have been made to surrogate the histopathological prognostic factors with clinical features. Based on the results of the 10-year Collaborative Ocular Melanoma Study (COMS), older age and larger maximal basal diameter were associated with metastasis as a cause of death [11]. Recently published data showed combining genetic information (TCGA groups) and information on tumor size and extraocular extension (AJCC stage) yields better prognostication in patients with uveal melanoma [12].

To the best of our knowledge, there is no published report to date comparing Ruthenium-106 plaque radiotherapy treatment in patients with small to medium-sized (< 7 mm in thickness) versus large (≥ 7 mm) UM.

Herein, we report two decades of visual and anatomical outcomes based on the initial thickness, largest basal diameter, ciliary body involvement, and peripapillary location of the tumor in 234 UM tumors from 234 patients who were treated with Ru-106 plaque radiotherapy between 2003 and 2019 at a single referral center.

## Methods

The study protocol was approved by the local Ethics Review Committee of Iran University of Medical Sciences, and all participants provided written informed consent prior to inclusion. The study was conducted in accordance with the tenets of the declaration of Helsinki.

### Study participants

In this retrospective cohort study, medical records of all patients with ciliary body or choroidal melanoma treated with Ru-106 plaque radiotherapy at the Ocular Oncology Service, Rasoul Akram Hospital, Iran University of Medical Services, Tehran, between November 1, 2003 and September 31, 2019, were reviewed. All patients underwent ophthalmic examination, including slit lamp biomicroscopy, indirect ophthalmoscopy, and ophthalmic imaging with fundus photography, ultrasonography, fundus autofluorescence, optical coherence tomography (OCT), and fluorescein angiography (when indicated) at the initial visit and every 4 months in the following first 2 years after plaque radiotherapy and every 6 months thereafter.

### Data collection

The data were collected at each examination. In addition to imaging documents and the demographic features of age, sex, and study eye, the clinical features collected at the initial examination included best-corrected visual acuity (BCVA) and tumor features, i.e., distance (in millimeters) to the optic nerve, distance to foveola, largest basal diameter (in millimeters), tumor thickness (in millimeters), and tumor location. All measurements were based on disc diameter or binocular indirect ophthalmoscopy estimations. The tumor stage was determined using the American Joint Committee on Cancer classification of uveal melanoma, eighth edition [13]. The plaque radiotherapy features included treatment duration, plaque shape (CCA, CCB, CGD, COB, CIA types [Eckert & Ziegler BEBIG, Berlin, Germany]), and total dose (Gray [Gy]) and dose rate (Gray per hour) to tumor apex and sclera.

Clinical outcomes included follow-up time, best-corrected visual acuity, Subretinal fluid evidence, final tumor basal diameter and thickness, development of OCT-evident cystoid macular edema (CME), clinically evident radiation retinopathy, radiation maculopathy, radiation papillopathy, neovascular glaucoma, cataract, scleral necrosis, distant metastasis, and death. Radiation retinopathy was defined as either non-proliferative occlusive microangiopathy manifesting as any (or combinations) of retinal hemorrhage, telangiectatic vessels, cotton-wool spots, microaneurysm, and macular exudate and or proliferative retinopathy associated with neovascularization [14]. Radiation maculopathy was defined as a 20% or more increase in central retinal thickness on OCT in comparison to the fellow eye. The presence of peripapillary nerve fiber layer infarction, optic disc edema, or pallor were considered as radiation papillopathy [15].

### Protocol of treatment

**Fig. 1:**
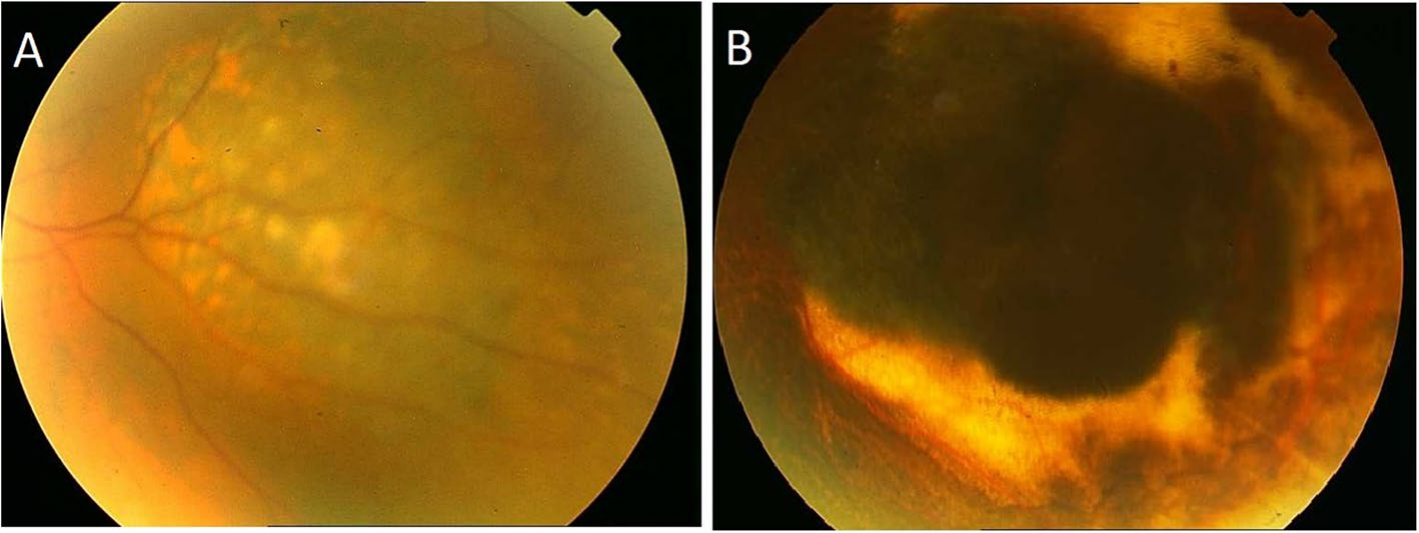
Fundus photo of a patient with choroidal melanoma before treatment (**A**) and one year after plaque therapy and adjuvant TTT (**B**)

All surgeries were performed by a single surgeon (MN) under general anesthesia. In small or medium-sized tumors, the target dose to the apex was considered 100 Gy. However, for thick tumors (> 7 mm) in which the tumor apex will receive less than a 100 Gy target dose, our protocol for Ru-106 plaque radiotherapy permits a maximal scleral dose of 1500 Gy [16, 17]. The type of radioactive plaque was selected based on the location and dimensions of the tumor. The Ru-106 plaques were supplied by BEBIG Company (BEBIG Isotopen und Medizintechnik GmbH, Berlin, Germany). The clinical target volume (CTV) was based on LBD plus 2 mm and an extra 1 mm to apical tumor height to compensate for 1 mm thickness of sclera. Isodose planes perpendicular and parallel to the central axis of the plaques were used to assessed the CTV coverage. Considering a safety margin of 2 mm, CCA, CCB, and CGD plaques were usually used for tumors with a base diameter (LBD) up to 11, 16, and 18 mm, respectively. Notched (COB) and CIA plaques were used for tumors adjacent to the optic nerve and ciliary body lesions, respectively. During the surgery, the margin of pigmented and non-pigmented tumors was delineated via transillumination and binocular indirect ophthalmoscopy. Acrylic dummies were temporarily sutured to the sclera to indicate the location of Ru-106 plaque, and extraocular muscles were disinserted using hang-backed sutures if necessary. Patients were evaluated by slit lamp examination, fundoscopy (Fig. 1), and B-scan imaging at 2 weeks and at 1, 2, 4, and 6 months after surgery. In the case of insufficient response (increase in size as well as persistent subretinal fluid 6 months after the brachytherapy in the absence of retinal hole or break) or recurrence (i.e., increase in size and/or thickness), secondary plaque insertion, transpupillary thermotherapy (TTT), or enucleation was considered for management of the tumor. In addition to residual subretinal fluid, equivocal evidence of shrinkage and lack of increased echogenicity in the ocular B-scan were further indications for performing adjuvant TTT. All patients were assessed by liver ultrasonography and liver function tests biannually for early detection of metastasis. Suspicious lesions in the liver were assessed by triphasic CT-scan or contrast-enhanced MRI. In selected suspicious cases, the oncologist performed a liver biopsy to rule out metastasis. The patients were then scheduled for regular follow-up every 6 months. In the case of deceased patients, the cause of death was retrieved from medical records. When follow-up did not occur, contact was made with the patient or his/her family via telephone if possible. If not, a censor was given at the next follow-up visit. Patients with less than 6 months of follow-up were excluded from the study.

### Statistical analysis

Statistical analysis was performed using SPSS software (version 22 for Windows, IBM, Armonk, NY, USA). Continuous variables were expressed as mean ± SD (median, range), and categorical variables were expressed as n (%). One sample Kolmogorov-Smirnov test was used to assess the normal distribution of variables in our sample, and logarithm transformation of the variables without normal distribution was used in our regression models. The results were analyzed based on thickness (thick tumors with a thickness ≥ 7 mm and thin tumors with a thickness < 7 mm), LBD (large tumors with LBD > 12 mm and small/medium tumors with LBD ≤ 12 mm), tumor location (juxtapapillary and ciliary body involvement location vs. elsewhere). Comparisons between the two groups were performed using a student independent sample *t*-test for continuous variables with normal distribution and a Mann-Whitney U test for continuous variables without normal distribution. Before and after comparisons were performed using a paired-samples *t*-test for continuous variables with normal distribution and a Wilcoxon test for continuous variables without normal distribution. Kaplan-Meier analysis was used for the estimation of globe survival, metastasis, and death. Cox-regression analysis was implemented for the evaluation of factors affecting survival. A *p-*value less than 0.05 was considered statistically significant, and all *p*-values were 2-sided.

## Results

### Demographic data and tumor characteristics

A total of 234 tumors from 234 patients were included in the study. The mean age of the patients at diagnosis was 51.86 ± 14.28 years. One-hundred-thirty-six (58.1%) patients were female and 98 (41.9%) were male. The mean ± SD of LBD and tumor thickness was 12.11 ± 3.74 mm and 6.01 ± 2.17 mm, respectively. The rate of decrease in thickness was 30 and 45% at 6 months following treatment and the last visit, respectively. Baseline clinical characteristics of the tumors are presented in Table 1. The mean ± SD of the optic nerve head overhanging was 20 ± 32% in eyes treated with notched plaque. Inferior oblique (IO) myectomy and rectus muscle disinsertion were performed in 11 (4.7%) and 17 (7.3%) patients, respectively. Treatment parameters are summarized in Table 2. Comparison of the baseline and final characteristics of the tumors are shown in Table 3.

**Table 1:** Baseline clinical features of the tumors

Tumor location	
Choroidal melanoma	186 (79.5%)
Ciliochoroidal melanoma	48 (20.5%)
Tumor location	
Juxtapapillary	46 (19.7%)
Elsewhere	188 (80.3%)
Tumor shape	
Mushroom shaped	35/186 (18.8%)
Dome shaped	151/186 (71.2%)
Tumor dimensions	
largest base (mean, range), mm	(12.1, 4–20)
Width (mean, range), mm	(10.1, 3–18)
Thickness (mean, range), mm	(6.0, 2.5–11)
Distance to optic disc, mm	4.12 ± 3.12
Distance to fovea, mm	4.24 ± 3.05
Basal diameter	
> 12 mm	109 (46.6%)
≤ 12 mm	125 (53.4%)
Thickness	
≥ 7 mm	86 (36.8%)
< 7 mm	148 (63.2%)
Staging	
T1	26.5%
T2	38.9%
T3	26.5%
T4	8.1%

**Table 2:** Radiation Parameters

Radiation hours (mean, range)	135 (31–314)
Apex dose rate (Gy/h) (mean, range)	0.94 (0.13–3.2)
Apex dose (Gy) (mean, range)	86 (28–110)
Scleral dose rate (Gy/h) (mean, range)	6.1 (2.1–10.1)
Scleral dose (Gy) (mean, range)	803 (170–1493)
Notched plaque	46 (28.2%)

**Table 3:** Baseline tumor features vs. final visual and anatomical control outcomes

Parameter	Baseline	Final	*P* value^*^
Largest Basal Diameter (Mean ± SD)	12.11 ± 3.74 mm (range: 4 to 20 mm)	9.95 ± 3.42 mm (2.5 to 20 mm)	< 0.001
Thickness (Mean ± SD)	6.01 ± 2.17 mm (range: 2.5 to 11 mm)	3.53 ± 2.07 mm (0 to 10 mm)	< 0.001
Visual acuity (logMAR)	0.673 ± 0.70	0.963 ± 0.80 ^ϯ^	< 0.001
` > 20/200	60.3%	44.2%	
> 20/40	28.6%	18.1%	

* paired-samples T-test; ^ϯ^ excluding enucleated eyes

### Globe survival

**Fig. 2:**
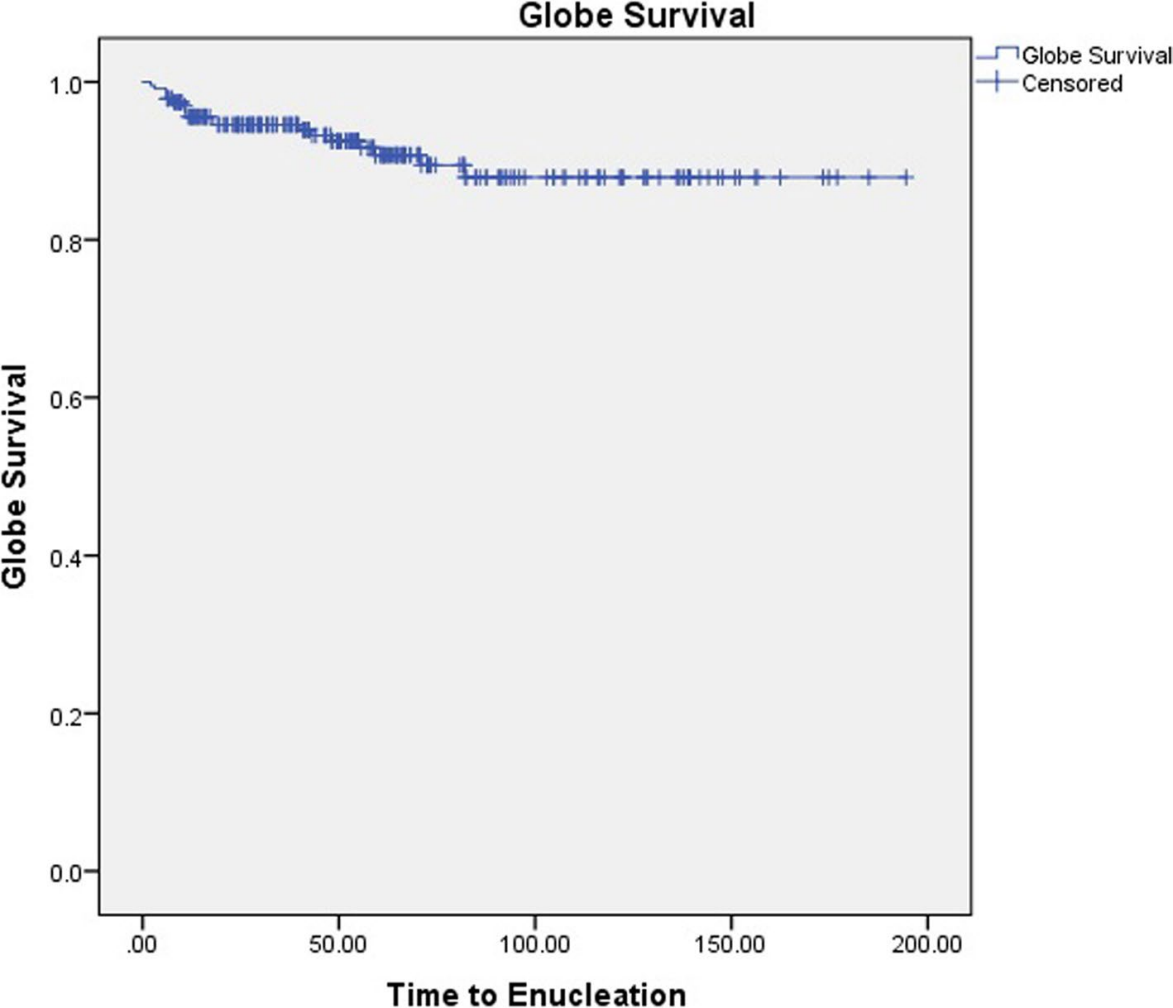
The Kaplan-Meier plot of globe survival

The patients were followed for an average of 61 ± 43 months (range 6–194.5 months). The anatomical success rate was 91.9%. Based on the Kaplan-Meier analysis, globe survival was estimated to be 0.879 (Fig. 2). Recurrence was observed in 30 patients (12.8%), which was managed by enucleation in 19 patients (63.3%) and by secondary plaque and TTT in 11 patients (36.6%). Adjuvant TTT was performed in 88 patients (35.5%). Tumors with recurrence had a greater baseline thickness (6.76 ± 2.31 mm vs. 5.91 ± 2.14 mm, *p* = 0.045) and received a smaller radiation dose to apex (75.9 ± 25.8 Gy vs. 87.0 ± 20.8 Gy, *p* = 0.010).

However, there was no significant difference between thick vs. thin tumors regarding the tumor recurrence rate (17.4% vs. 10.1% *p* = 0.107) and anatomical success rate (90.7% vs. 92.6%, *p* = 0.614). In addition, the LBD grouping and ciliary body involvement (Table 4) did not affect the recurrence and enucleation rate. On the other hand, eyes treated with COB (juxtapapillary tumors) had a higher rate of enucleation (19.6% vs. 5.3%, *p* = 0.002) with no difference in the recurrence rate (Table 5). In juxtapapillary tumors, there was no statistically significant difference in the rate of enucleation of the tumors with or without overhanging (25% vs. 16.7%, *p* = 0.277). In addition, there was no statistically significant association between TNM staging (T-categories) and the rate of enucleation in the Cox regression analysis (enucleation rate of 8.1% in T1, 5.5% in T2, 11.3% in T3, and 10.5% in T4, *p* = 0.249).

**Table 4:** Comparison between tumors with and without ciliary body involvement

	With ciliary body involvement	Without ciliary body involvement	*P*-value
Number	48	186	
Thickness (mean,range)			
Baseline	6.6 (3–11) mm	5.9 (2.5–11) mm	0.025
Final	4.7 (0.5–6.5) mm	3.3 (0.0–8.0) mm	0.000
Visual acuity			
Baseline	0.63 logMAR	0.70 logMAR	0.580
Final	0.93 logMAR	0.97 logMAR	0.766
Recurrence	7 (14.6%)	23 (12.4%)	0.682
Enucleation	5 (10.4%)	14 (7.5%)	0.513
Metastasis	6 (12.5%)	10 (5.4%)	0.081

**Table 5:** Comparison between Notched plauqe and other types of plaque

	Notched Plaque	Other Plaques	*P*-value
Number	46	188	
Thickness (mean,range)			
Baseline	6.2 (2.5-11) mm	5.3 (2.5-9.5) mm	0.010
Final	3.1 (1.0-6.5) mm	3.4 (0.0-8.0) mm	0.324
Visual acuity			
Baseline	0.84 logMAR	0.64 logMAR	0.111
Final	0.88 logMAR	0.98 logMAR	0.527
Recurrence	9 (19.6%)	21 (11.2%)	0.127
Enucleation	9 (19.6%)	10 (5.3%)	0.002
Metastasis	2 (4.3%)	14 (7.4%)	0.455

In multivariable Cox regression analysis of different analysis groups, the hazard ratio (HR) for enucleation was only statistically significant for juxtapapillary tumors (HR = 6.58, 95-CI: 3.84 to 11.21, *p*-value = 0.000).

### Metastasis and patient survival

**Fig. 3:**
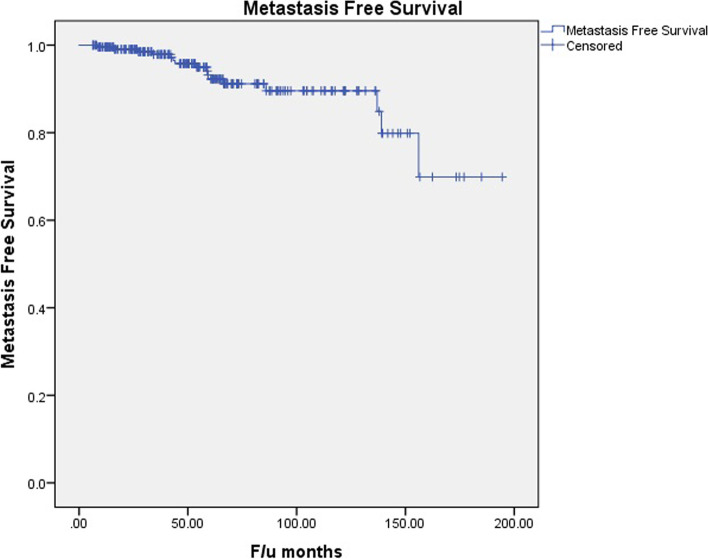
The Kaplan-Meier plot of metastasis-free survival

The rate of metastasis in this cohort was 6.8% (16 patients) with a mean interval of 38.2 ± 18.7 months (range:14 to 57 months) between initial diagnosis of the tumor and metastasis. Based on the Kaplan-Meier analysis, the actuarial metastasis-free survival rates were estimated to be 0.99 and 0.95 at 5 years and 10 years, respectively (Fig. 3).

Up until the completion of current study, of patients with metastasis, 81.25% (13 of 16 patients) died with a mean interval of 7.2 ± 3.7 months after detection of metastasis. In the Cox regression analysis, the occurrence of metastasis had a marginally significant association with tumor thickness (B = 0.227, 95-CI: 0.111 to 0.343, *p*-value = 0.050), and there was no significant association between recurrence of the tumor (*p*-value = 0.662) or TNM staging (*p*-value = 0.32) and the incidence of metastasis. In addition, there was no statistically significant difference between metastasis rate in thick and thin tumors (8.1% vs. 6.1%, *P* = 0.548), large and small/medium tumors (10.1% vs. 4.0%, *P* = 0.066), and different locations of the tumor.

### Radiation complications

Regarding radiation complications, 108 eyes (46.2%) developed cataracts following brachytherapy. The rate of radiation retinopathy at the last follow-up was 58.1%. Moreover, 70 (29.9%) and 47 eyes (20.1%) developed radiation maculopathy and radiation papillopathy, respectively. In the logistic regression analysis, radiation papillopathy was associated with a total scleral dose (B = 0.001, 95% CI: 0.001 to 0.001, *p*-value = 0.036) and distance to the optic nerve head (B = -0.292, 95% CI: − 0.226 to − 0.358, *p*-value< 0.001). Radiation maculopathy was only associated with distance to the fovea (B = -0.284, 95-CI: − 0.230 to − 0.338, *p*-value< 0.001).

Ten patients (4.3%) developed glaucoma. Four patients (1.7%) had neovascular glaucoma, which was managed by shunt surgery in 2 patients and led to enucleation in the remaining patients. Vitreous hemorrhage requiring pars plana vitrectomy developed in 19 eyes (8.3%). Scleral necrosis was observed in 5 patients (2.13%), all of whom were cases with ciliary body involvement with mean scleral dose of 714.9 (range: 487.2–942.7) Gy and were managed using a corneal patch graft.

After adjusting for baseline visual acuity and excluding enucleated cases, final visual acuity was associated with radiation maculopathy (*p*-value = 0.015) in the multivariable general linear analysis. The risk of legal blindness (visual acuity less than 0.1) was 44.8%.

## Discussion

In the present study, we showed that Ru-106 plaque radiotherapy is an effective treatment for both large and small/medium-sized UM with no difference in recurrence and anatomical success between the two groups in terms of tumor thickness (< 7 mm vs. ≥ 7 mm).

The 5-year globe survival in our study was 0.946, which is comparable to the literature regarding brachytherapy of small to medium choroidal melanoma with a reported enucleation rate of 4–5% [18, 19]. On the other hand, we found a relatively higher rate of recurrence (12.8%) compared to the 3% at 7 years found by Damato et al., [20] which could be attributed to the inclusion of tumors with a higher mean thickness (6.01 ± 2.17 mm) in our study. Similar studies with tumors of comparable size have reported a recurrence rate of 11–16% [21–23]. However, the higher recurrence in thinner tumors particularly in juxtapapillary choroidal melanomas, can be justified by marginal tumor recurrence which were managed by TTT in most of these patients.

In a previous study, we showed that contrary to established guidelines, thick choroidal melanomas are amenable to Ru-106 brachytherapy with a lower apex dose rate [8]. The results of the current study showed that although the rate of recurrence was higher in thick tumors vs. thin tumors after brachytherapy, this difference was not statistically significant and did not lead to lower globe preservation in thick tumors. In addition, based on our results, although the recurrence rate is associated with greater thickness and lower apex dose, they can be managed safely with secondary plaque or enucleation without increasing the metastasis rate, confirming that globe-preservation therapy does not impair the patient’s survival [24]. This is contrary to previous studies that showed higher rate of metastasis in recurrent cases [25]. In addition, different rate of metastasis is reported for distinct patterns of local recurrence as higher chance of metastasis is observed in cases of vertical recurrence vs. marginal. The overall low rate of metastasis in our study and not assessing the recurrence pattern might justify this difference.

The local control rate in juxtapapillary UM has been reported to be 90–94.1% with iodine-125 notched plaque brachytherapy [26, 27], which is higher than the 80.4% in our study. In addition, the multivariable Cox regression analysis showed that the hazard ratio was statistically significant for the peripapillary location of the tumor. This difference could be attributed to the custom shape of iodine-125 plaques in comparison to the fixed shape of COB Ru-106 plaques, which might not entirely encompass the tumor.

After adjusting for baseline visual acuity, the final logMAR BCVA was associated with radiation maculopathy and papillopathy, and the rate of legal blindness (BCVA < 20/200) was 44.8%, which is comparable to other large studies reporting a 52–55% rate of retaining functional visual acuity (BCVA > 20/200) [19, 28, 29]. UM is relatively radioresistant. Therefore, brachytherapy for these tumors is inherently associated with high rates of radiation complications and subsequent damage to the optic nerve and macula. Apart from the acute exudative complications of radiation, the slowly progressive vaso-occlusive disease will continue to deteriorate visual acuity many years after initial treatment [19]. It should be noted that apart from radiation complications, the loss of VA following plaque radiotherapy depends on several factors, including initial visual acuity, the temporal location of the tumor, distance to the fovea and optic nerve head, concurrent systemic diseases, and age of the patient.

Similar to previous studies [30, 31], we found a strong association between the development of radiation-related complications and scleral dose rate and the shorter distance of tumor margin to the macula and optic nerve head.

Neovascular glaucoma developed in 1.7% of our patients, similar to the 1% rate of this complication in ^125^I plaque radiotherapy [18] and the 1.9% rate in Ru-106 plaque therapy [32]. However, our rate is much lower than the 12–31% rate of neovascular glaucoma associated with proton beam radiotherapy [33]. This lower rate is mainly due to the toxic tumor syndrome secondary to helium ion irradiation for large uveal melanomas [34].

Extraocular extension (EOE) is a relatively rare complication in small to medium-sized tumors and is usually observed in ciliary body tumors [35]. In the current study, the rate of EOE was zero; however, post-radiation scleral necrosis was observed only in patients with ciliary body involvement at mean scleral dose of 714.9 (range: 487.2–942.7) Gy. Based on our previous experience [36], enucleation could be avoided in these cases by performing corneal patch graft without an obvious increase in the risk of subsequent metastasis. It should be noted that we consider the 1500 Gy as the safe dose for sclera and studies with lower threshold for scleral dose reported a 1% rate for scleral necrosis [37].

Metastasis occurred in 6.8% of the patients in this study, which is significantly lower than similar studies [19, 38, 39]. This rate might reflect the different biology of UM in Iranian patients. It has been shown that monosomy 3, as a poor prognostic factor, has a low incidence rate (8%) in enucleated eyes with choroidal melanoma in Iranian patients [40]. Interestingly it has been reported that the rate of metastasis is lower in the Asian population [41], especially in middle eastern countries such as Saudi Arabia (metastasis rate of 5%) [42] and Jordan (metastasis rate of 13%) [43], which might signify the role of ethnicity in survival from uveal melanoma. In addition, excluding large UM tumors that were candidates for primary enucleation probably affected the rate of metastasis. Gene expression profiling has shown that class 1 tumors associated with lower risk metastasis have a high rate of decrease in tumor thickness up to 6 months (26.8%) following brachytherapy [44]. Our patients exhibited a similar decrease of 30% in tumor thickness at 6 months following brachytherapy, which might be due to the higher prevalence of class 1 tumors in our patients. Another possible explanation is the limited follow-up time in our study. Based on COMS studies, the rate of metastasis increases 10 years after the initial diagnosis of an ocular tumor [45]. Moreover, despite the established association between the rate of metastasis and an increase in tumor thickness [46], the low rate of metastasis in our study could justify the finding of non-significant association. It should be noted that despite the low rate of metastasis in our study, patients experiencing metastasis had a poor prognosis with high mortality 7 months following the detection of metastasis, similar to the available literature reporting overall survival of 1.07 years [47].

Our study has some limitations. First, the relatively limited follow-up time might affect the detection of metastasis in the current cohort. Second, the retrospective nature of the study and the lack of real-time access to radioactive plaques during some periods may be associated with lower dose rates, which would influence the obtained results. In addition, due to some restrictions we were forced to used old plaques for few patients which resulted in prolonged admission therefore, we suggest using “hot” Ru-106 plaques for thicker tumors.

In conclusion, although iodine-125 plaques versus enucleation are generally recommended for thicker uveal melanomas, in case of unavailability of this isotope, Ru-106 plaque brachytherapy can be a treatment option in patients diagnosed with uveal melanoma with a thickness up to 11 mm who refused the enucleation with no significant difference in tumor control rate, metastasis, and patient survival in thick and large tumors (≥ 7 mm in thickness and > 12 in LBD) compared to thin and small/medium tumors. In addition, the globe salvage rate is lower with notched Ru-106 plaque for juxtapapillary tumors in comparison to the other types of Ru-106 plaques for elsewhere.

## Data Availability

The datasets used and/or analysed during the current study are available from the corresponding author on reasonable request.
